# Single-cell RNA sequencing revealed cell landscape of tongue dorsal mucosa in rats with gastric intestinal metaplasia

**DOI:** 10.1038/s41420-025-02386-z

**Published:** 2025-03-16

**Authors:** Jiao Xiang, Jing Han, Jianping Wu, Shuo Xu, Chun Cheng, Junfeng Zhang

**Affiliations:** 1 School of Medicine, Nanjing University of Chinese Medicine, 210023 Nanjing, Jiangsu China; 2 Laboratory Animal Center, Nanjing University of Chinese Medicine, 210023 Nanjing, Jiangsu China

**Keywords:** Gastritis, Gastroenteritis

## Abstract

The formation of tongue coating is closely related with the differentiation of the lingual dorsal mucosa, and a great deal of evidence shows that the variation of tongue coating reflects the pathological and physiological state of the gastric mucosa. However, the detailed mechanism remains elusive. This study established a rat model of gastric intestinal metaplasia (GIM) with 2% sodium salicylate and 20 mmol/L of deoxycholate sodium, and used single-cell RNA sequencing (scRNA-seq) to reveal the cell landscape of tongue dorsal mucosa. In comparison to the control group, the tongue dorsal mucosa of GIM rats became grayish-white, and the histologic characteristics presented an uneven distribution of tongue papilla with many immune cells in the submucosal layer. The expressive levels of pro-inflammatory factors (IL-1β, IL-6, and IL-17) were significantly higher in GIM rats than in the control group. Stratified analysis revealed the significant downregulation of autophagy marker gene *Map1lc3a* in neutrophils and T cells, and the significant downregulation of cuproptosis marker gene *Dlst* in fibroblasts of the tongue dorsal mucosa in GIM rats. These changes were closely related to mucosal inflammation and impaired tissue barrier integrity. Significantly, the expression of several keratin genes (*Krt7*, *Krt8*, *Krt13*, *Krt16*, and *Krt76*) was significantly downregulated, as well as the expression of the bitter taste receptor gene *Rtp4* and the sweet taste receptor gene *Tas1r2* in the GIM rats. The data indicated that fewer cells entered regulated cell death in immune cells of tongue mucosa, a more active inflammatory response occurred, the keratinization of tongue dorsal mucosal cells was inhibited, and the taste perception function was weakened. The results bring new perspectives on tongue coating in the application of gastric disorders.

**Figure:**
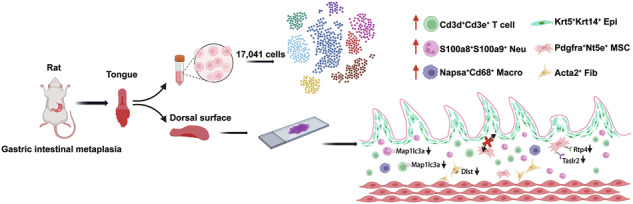
Characteristics of the tongue dorsum mucosal cell landscape in the rats with gastric intestinal metaplasia. The abundances of T cells, neutrophils, and macrophages were upregulated, and the autophagy marker gene *Map1lc3a* in T cells and neutrophils was downregulated, which indicated an actively inflammatory immune response. Downregulation of cuprotosis marker gene *Dlst* in fibroblasts suggested potential damage to the mucosal barrier. Meanwhile, the expression of bitter receptor Rtp4 and sweet receptor Tas1r2 in mesenchymal stem cells was downregulated. The cell communication ability was reduced, especially between mesenchymal stem cells and epithelial cells. In a word, the abnormal status of tongue dorsum mucosa may accompany the development of gastric intestinal metaplasia.

Characteristics of the tongue dorsum mucosal cell landscape in the rats with gastric intestinal metaplasia. The abundances of T cells, neutrophils, and macrophages were upregulated, and the autophagy marker gene *Map1lc3a* in T cells and neutrophils was downregulated, which indicated an actively inflammatory immune response. Downregulation of cuprotosis marker gene *Dlst* in fibroblasts suggested potential damage to the mucosal barrier. Meanwhile, the expression of bitter receptor Rtp4 and sweet receptor Tas1r2 in mesenchymal stem cells was downregulated. The cell communication ability was reduced, especially between mesenchymal stem cells and epithelial cells. In a word, the abnormal status of tongue dorsum mucosa may accompany the development of gastric intestinal metaplasia.

## Introduction

Gastric intestinal metaplasia (GIM) is a typical precancerous lesion of gastric cancer (GC), characterized by the replacement of epithelial cells by Paneth cells, goblet cells, and intestinal absorptive cells [1]. Gastroscopy and pathology are the gold standard for diagnosing GIM, but the fear of gastroscopy always hinders the step of early diagnosis and treatment of GIM, which causes a high incidence of GC-related death. Many clinical evidences have indicated the close linkage between oral status and gastric disorders, especially the cross-talk between oral and gastric microbiota [2], which indicates that the oral cavity can provide non-invasive biomarkers for diagnosing gastric disorders besides GIM and GC. In other words, the oral cavity serves as a window to human health and diseases.

The tongue is an important organ in the oral cavity for the functions of food mixing, aiding in pronunciation, and taste sensing. Tongue diagnosis in traditional Chinese medicine (TCM) boasts a long history, and TCM theory holds that observation of tongue coating can speculate the function of Spleen–Stomach, which generally refers to the digestive function [3]. Mounting evidence has shown that the tongue-coating microbiota has potential diagnostic value in system disorders, especially in upper gastrointestinal disease [4]. For instance, Xu et al. presented the promising value of tongue-coating microbiota as a non-invasive biomarker for diagnosing GC [5]. Chen et al. found tongue *Schaalia odontolytica* was associated with higher GC risk, while *Acetatifactor* was related to lower GC risk [6]. Cui et al. proved the positive correlation between the abundance of *Campylobacter concisus* and the stage of GC development [7]. Shang et al. found that the specific tongue features (cyanotic tongue, slippery fur, yellow fur, tooth-marked tongue, and spotted tongue) linked closely with certain gastric pathological statuses (gastric antrum mucosal hyperemia, edema, erythema, and macula) in patients with chronic gastritis [8]. The latest clinical investigation demonstrated an artificial intelligence deep learning model based on tongue images, tongue coating microbiota, and blood biomarkers in diagnosing GC [9]. These findings suggest that tongue coating reflects the gastric mucosal lesions, but the detailed biological mechanism remains mysterious.

The formation of tongue coating depends on the proliferation, differentiation, migration, and apoptosis of epithelial cells [10]. Early studies found that rats with chronic atrophic gastritis (CAG), induced by 2% sodium salicylate and 20 mmol/L of deoxycholate sodium, exhibited obvious alternation of tongue dorsal mucosa, including thickening of stratum corneum, dysplasia of filiform papillae, and manifold nuclear vacuolation in the granular layer [11]. Furthermore, in vitro experiments confirmed that the apoptotic pattern of tongue epithelial cells of rats is similar to that of humans [12]. However, the detailed cell landscape of tongue coating is still unclear.

In recent years, the emerging single-cell RNA sequencing (scRNA-seq) has laid a solid foundation for the high-resolution characterization of cellular landscapes and functional features of tissues and organs. Wang et al. found that the tongue epithelial cells express many receptors binding coronaviruses and influenza viruses, and various pattern recognition receptors [13], which provided molecular evidence to bridge the correlation between tongue coating features and tongue microbiota. However, the impact of diseases on the cell landscape and function of tongue dorsal mucosa has not been reported.

Therefore, this study utilized the rat model with GIM and presented the cell landscape of the tongue dorsal mucosa using scRNA-seq. Here, preliminarily revealed the expression pattern of keratinization, taste, and death-related genes in the tongue dorsal mucosa, which would provide cell-level evidence for the application of observation tongue coating in assisting diagnosis and treatment of gastric precancerous lesions.

## Results

### Cell landscape of tongue dorsal mucosa in GIM rats

**Fig. 1:**
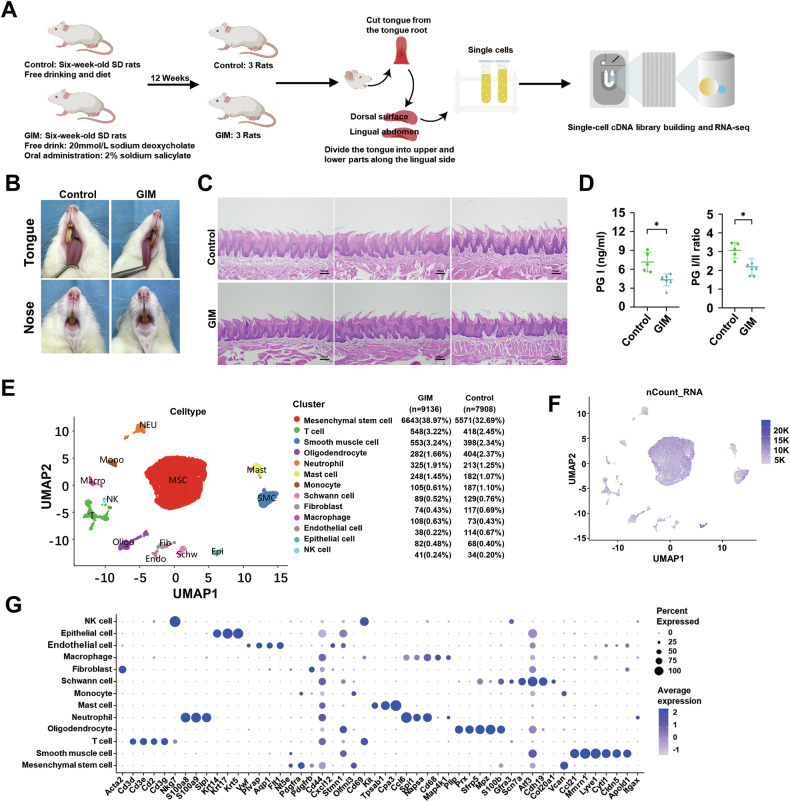
Cell landscape of tongue dorsal mucosa in rats with GIM. **A** The framework of this study. **B** The appearance of the tongue and nose in rats. **C** H&E staining showed the histological alteration of tongue dorsal mucosa in GIM rats (magnification: ×10, scale bar: 100 μm). **D** Serum PG I and PG I/II ratios were significantly decreased in GIM rats. **E** UMAP analysis reveals 13 cell clusters. **F** Transcriptional abundances of the cell clusters. **G** The signature genes of the cell clusters. Data with error bars are shown as mean ± standard deviation. *P < 0.05 as determined by independent *t*-tests.

The workflow for scRNA-seq of the tongue dorsal mucosa in GIM and control rats were shown in Fig. 1A. The results showed that the lips and noses of the control rats were ruddy, whereas those of the GIM rats appeared pale and dull; the lingual mucosa of the control was thin, white, and moist, while that of the GIM rats had a pale appearance (Fig. 1B). Histological results showed that the tongue dorsal mucosa of GIM rats presented uneven distribution of papillae, along with inflammatory cell infiltration in the stratum basal (Fig. 1C). Serological analysis showed that the gold standard of gastric mucosal atrophy, pepsinogen (PG) I and PG I/II ratio in GIM rats, were significantly lower than in the control (*P* < 0.05) (Fig. 1D). The morphological and histological results confirmed that the tongue dorsal mucosa was significantly altered in the rats with GIM.

A total of 17,044 cells were obtained from the tongue dorsal mucosa, including 7908 cells in the control and 9136 cells in GIM rats. The cells were clustered into 13 clusters, which were mesenchymal stem cells (MSC), T cells (T), smooth muscle cells (SMC), oligodendrocytes (Oligo), neutrophils (Neu), mast cells (Mast), monocytes (Mono), schwann cells (Schw), fibroblast (Fib), macrophage (Macro), endothelial cells (Endo), epithelial cells (Epi) and natural killer cells (NK) (Fig. 1E). Based on gene expression and principal component analysis (PCA), the distribution of cellular transcripts was shown in Fig. 1F. Thirteen cell types were defined by marker genes (Fig. 1F, Table S1), including MSC (*Nt5e*, *Pdgfra*), T (*Cd3d*, *Cd3e*, *Cd3g*), SMC (*Ccl21*, *Mmrn1*, *Lyve1*), Oligo (*Pllp*, *Prx*, *Sfrp5*), Neu (*S100a8*, *S100a9*), Mast (*Kit*, *Tpsab1*), Mono (*Vcan*), Schw (*Scn7a*, *Cdh19*), Fib (*Acta2*), Macro (*Cd68*), Endo (*Vwf*, *Plvap*, *Aqp1*), Epi (*Krt5*, *Krt14*, *Krt17*), and NK (*Nkg7*).

Ptprc is known as CD45 antigen and is expressed exclusively by leukocytes. In the tongue dorsal mucosa of rats, the percentages of CD45-positive cells in the control group and the GIM group were 7.7% and 8.8%, respectively (Fig. S1A), and the expression level of *CD45* in the tongue dorsal cells of GIM rats was significantly higher than that of the control group (*P* < 0.05) (Fig. S1B). Further cell cycle analysis showed significant changes in the distribution of T, Epi, Macro, NK, and Schw in the tongue dorsal mucosa of GIM rats. Of them, T cells showed G1 phase proportions of 5.74% and 12.96%, with corresponding G2/M phase proportions of 76.32% and 67.88% in control and GIM rats, respectively. Similarly, G1 phase proportions of Epi were 38.24% and 25.61%, and G2/M phase proportions were 45.59% and 57.32%; S phase proportions of Macro were 21.92% and 10.18%, and G2/M phase proportions were 50.68% and 62.04%; S phase proportions of NK were 47.06% and 19.52%, and G2/M phase proportions were 41.18% and 65.85%; G2/M phase proportions of Schw were 36.43% and 49.44%, and G1 phase proportions were 34.11% and 26.97% (Fig. S1C, Tables S2 and S3). The above data suggested that more immune cells appeared with significant alteration of cell cycle distributions in the dorsal mucosa of the tongue with GIM.

### Analysis of gene expression of keratins and taste receptors in tongue dorsal mucosa in rats with GIM

**Fig. 2:**
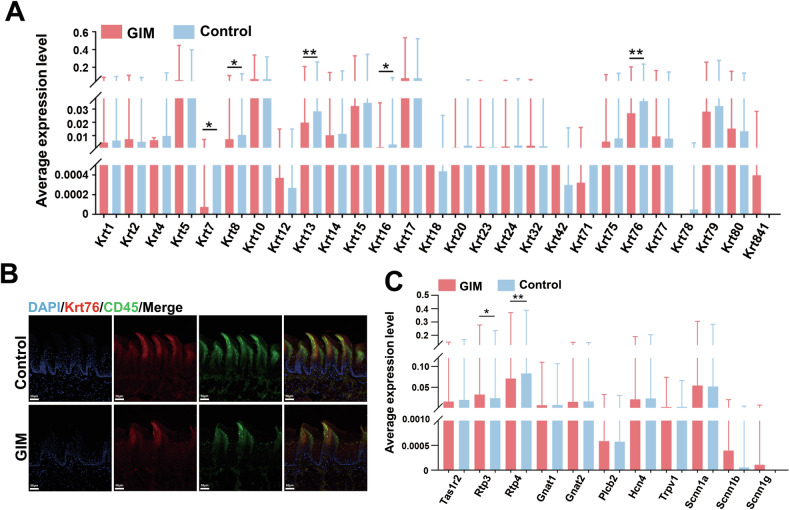
Analysis of keratin and taste receptor genes expressing in the tongue dorsal mucosa of GIM rats. **A** Differential analysis of 27 keratin genes in tongue dorsal mucosal cells between control and GIM rats. **B** Immunohistochemical staining verified the expression levels of Krt76 and CD45 in the tongue dorsal mucosa of GIM rats (magnification: ×20, scale bar: 50 μm). **C** Differential analysis of taste sensation-related genes in tongue dorsal mucosal cells between control and GIM rats. Data with error bars are shown as mean ± standard deviation. *P < 0.05; **P < 0.01 as determined by independent *t*-tests.

Keratinocytes are the predominant cells in the epidermis of the tongue dorsal mucosa. Keratins (Krts) exhibit highly specific expression patterns during the keratinization process of epithelial cells, participating in environmental adaptation, damage and repair, and local immune response [14]. In comparison with the control group, the expressive levels of five Krt genes (*Krt7*, *Krt8*, *Krt13*, *Krt16*, and *Krt76*) were significantly decreased in the tongue dorsal mucosa of GIM rats (Fig. 2A, Table S4). Stratified analyses revealed higher expression levels of Krts in MSC and Epi. In GIM rats, lower *Krt4* expression was observed in Epi, lower *Krt10* expression was observed in Oligo, and lower *Krt13* expression was observed in MSC in the tongue dorsal mucosa. Conversely, higher *Krt17* expression was observed in Fib (Fig. S1A, Table S5). Immunofluorescence staining verified the expressive levels of Krt76 and CD45 (Fig. 2B). It was noted that the higher expression of CD45 was observed in the papillary spaces and basal layer of the epithelium, while the lower expression of CD45 was observed at the upper part of tongue papillae, which suggested the ability of immune cells migrating towards the tongue papillae decreased in the tongue dorsal mucosa of GIM rats, indicating decreased mucosal defense function. In a word, the process of keratinization was inhibited in the tongue dorsal mucosal of GIM rats, and more inflammatory cells penetrated the basal layer but faced a challenge to exudate towards the mucosal surface.

Taste sensation is another function of tongue dorsal mucosa, the present study analyzed the expression of 11 gustatory receptor genes, and the bitter taste receptor genes, *Rtp3* (receptor transporter protein 3) was significantly upregulated, while *Rtp4* was significantly downregulated in GIM rats (Fig. 2C, Table S6). Stratified analysis revealed that upregulated *Rtp3* in SMC, downregulated *Tas1r2* (a sweet taste receptor gene, taste 1 receptor member 2), and *Rtp4* in MSC, were observed in the GIM rats (Fig. S2B, Table S7). The results indicated that the function of sensing bitterness and sweetness was weakened when the GIM occurred.

### Characterization of cell death in the tongue dorsal mucosa of GIM rats

The epithelial cells undergo continuous keratinization, shedding, and renewal, which are critical for maintaining a homeostatic balance of the tongue dorsal mucosa. According to previous literature, the well-known regulated cell death pathways include necroptosis, pyroptosis, and apoptosis [15]. In recent years, autophagy, ferroptosis and cuproptosis have garnered widespread attention [16, 17]. Therefore, this study focused on the six cell death pathways to explore the biological mechanism of the tongue epithelial cells in the GIM rats.

**Fig. 3:**
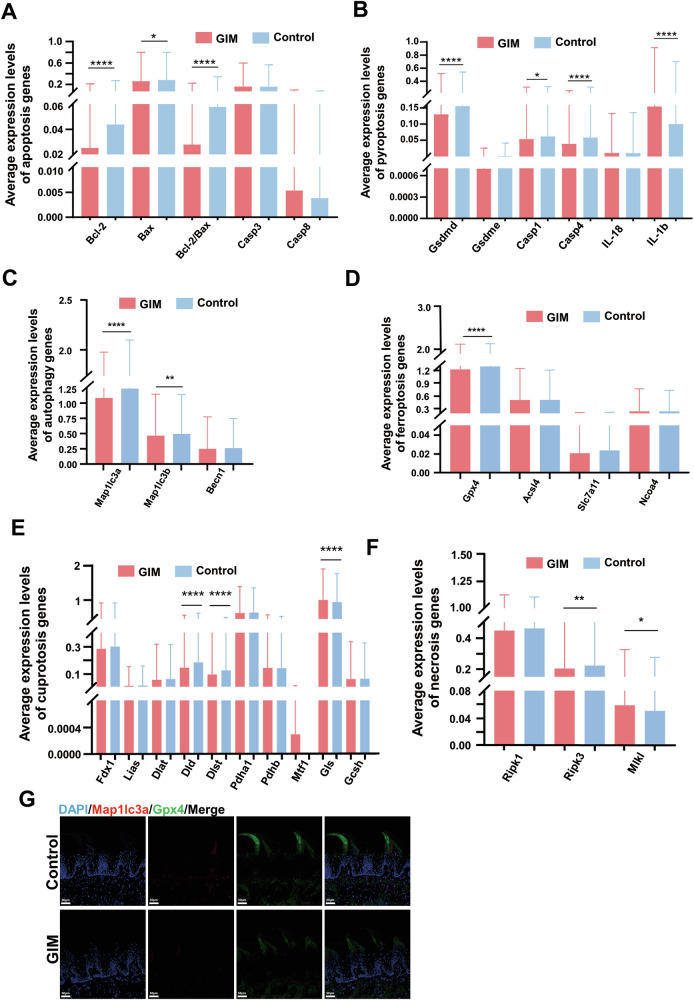
Characterization of cell death in the tongue dorsal mucosal tissue of GIM rats. Based on the cell-death-related marker genes, differential analysis was conducted between the control and GIM groups. **A** Differential analysis of apoptosis-related genes. **B** Differential analysis of pyroptosis-related genes. **C** Differential analysis of autophagy-related genes. **D** Differential analysis of ferroptosis-related genes. **E** Differential analysis of necroptosis-related genes. **F** Differential analysis of cuproptosis-related genes. **G** Immunofluorescence staining of Mapllc3a and Gpx4 expression in tongue dorsal mucosal tissue (magnification: ×20, scale bar: 50 μm). Data with error bars are shown as mean ± standard deviation. *P < 0.05, **P < 0.01, ***P < 0.001, and ****P < 0.0001 as determined by independent *t*-tests.

Firstly, the marker genes of apoptosis include *Bcl-2*, *Bax* and Cysteinyl aspartate-specific proteinase 3 (*Caspase3*). Compared with the control group, the expression of *Bcl-2* and *Bax* in the tongue dorsal mucosa of GIM rats were significantly decreased, along with the significant decrease of *Bcl-2* / *Bax* ratio, suggesting that the apoptosis was promoted in the tongue dorsal cells of GIM rats (Fig. 3A, Table S8). Stratified analysis showed that the downregulated *Caspase3* was observed in macrophages, downregulated *Bax* was observed in T cells, and downregulated *Bcl-2* was observed in MSC and oligodendrocytes (Fig. S3A, Table S9). The results suggested that apoptosis was weakened mainly in the immune cells of the tongue dorsal mucosa when GIM occurred.

Secondly, the marker genes of pyroptosis include cysteinyl aspartate specific proteinase-1 (*Caspase-1*), cysteinyl aspartate specific proteinase-4 (*Caspase-4*), gasdermin D (*Gsdmd*), and interleukin-1β (*IL-1β*). Compared with the control group, the expressive levels of *Caspase-1*, *Caspase-4*, and *Gsdmd* were significantly reduced in the tongue dorsal mucosa of GIM rats, while the expression of *IL-1β* was significantly increased (Fig. 3B, Table S8). Stratified analysis showed that the expressive levels of *Caspase-1*, *Caspase-4*, and *Gsdmd* were significantly decreased in MSC, while the expression of *IL-1β* was significantly increased. The decreased *Gsdmd* was observed in macrophages, whereas increased *IL-1β* was observed in T cells and neutrophils (Fig. S3B, Table S10). These results suggested that pyroptosis was hindered in the tongue dorsal mucosa of GIM rats, and the tongue inflammatory response might be indifferent to pyroptosis.

Thirdly, *Map1lc3a* (microtubule-associated protein 1 light chain 3 alpha), *Map1lc3b*, and Beclin1(*Becn1*) are the three famous marker genes of autophagy. The present study found that the expressive levels of *Map1lc3a* and *Map1lc3b* were significantly reduced in the tongue dorsal mucosa of GIM rats (Fig. 3C, Table S8), and immunofluorescence staining confirmed that the Map1lc3a was downregulated in the dorsal tongue papillae of GIM rats (Fig. 3G). Stratified analysis showed that downregulated *Map1lc3a* was broadly observed in MSCs, T cells, oligodendrocytes, neutrophils, mast cells, monocytes, Schwann cells, and endothelial cells; downregulated *Map1lc3b* was observed in MSCs and T cells (Fig. S3C, Table S11). The results indicated that autophagy was inhibited in the tongue dorsal mucosal cells of GIM rats, particularly in the immune cells such as T cells and monocytes, which was consistent with the infiltration of inflammatory cells in the basal layer of the tongue dorsal mucosa.

Fourthly, there are four well-known marker genes of ferroptosis, one is *Gpx4* (glutathione peroxidase 4) which inhibits ferroptosis, and the other three genes promote ferroptosis including *Acsl4* (long-chain acyl-CoA synthetase 4), *Slc7a11* (solute carrier family 7 member 11), and *Ncoa4* (nuclear receptor coactivator 4). Overall, the expression of *Gpx4* was significantly reduced in the tongue dorsal mucosa of GIM rats (Fig. 3D, Table S8), which was confirmed by immunofluorescence staining (Fig. 3G). Stratified analysis showed that the decreased *Gpx4* was observed in oligodendrocytes and NK cells, and the decreased *Acsl4* was observed in monocytes in the tongue dorsal mucosa of GIM rats (Fig. S3D, Table S12). The data indicated that the abnormal iron metabolism underwent in the tongue dorsal mucosa of GIM rats, and promoted oligodendrocytes and NK cells to ferroptosis.

Fifthly, necroptosis, a form of regulated cell death, is a gatekeeper of host defense against certain pathogen invasion, the marker genes include *Ripk1* (receptor-interacting protein kinase 1), *Ripk3* (receptor-interacting protein kinase 3), and *Mlkl* (mixed-lineage kinase domain-like protein). Overall, the expression of *Ripk3* was significantly decreased, while the expression of *Mlkl* was markedly increased in the tongue dorsal mucosa of GIM rats (Fig. 3E, Table S8). Stratified analysis showed that the reduced *Ripk3* was observed in neutrophils, macrophages, epithelial cells, and NK cells of GIM rats. The increased *Mlkl* was only observed in SMCs (Fig. S3E, Table S13). The necroptosis of immune cells and epithelial cells was simultaneously hindered in the tongue dorsal mucosa of GIM rats, which indicated that necroptosis might be linked with immune cell infiltration in the tongue dorsal mucosa of GIM rats.

Finally, eight genes have been identified for promoting cuproptosis, including *Fdx1* (ferredoxin 1), *Lias* (lipoic acid synthetase), *Dld* (dihydrolipoamide dehydrogenase), *Dlst* (dihydrolipoamide succinyltransferase), *Dlat* (dihydrolipoamide S-acetyltransferase), *Pdha1* (pyruvate dehydrogenase E1 subunit alpha 1), *Pdhb* (pyruvate dehydrogenase E1 subunit beta), and *Mtf1* (metal regulatory transcription factor 1). On the other hand, two genes have been identified for suppressing cuproptosis, including *Gls* (glutaminase) and *Gcsh* (glycine cleavage system protein H). Interestingly enough, the expressive levels of cuproptosis-promoting *Dld* and *Dlst* were significantly reduced, while the expression of cuproptosis-suppressing *Gls* was increased dramatically in the tongue dorsal mucosa of GIM rats (Fig. 3F, Table S8). Stratified analysis showed that decreased *Dld* and *Dlst*, and increased *Gls* were observed in MSCs. In addition, decreased *Dld* was observed in oligodendrocytes, decreased *Dlst* was observed in fibroblasts and macrophages, and decreased *Pdha1* was observed in monocytes (Fig. S3F, Table S14). These results indicated that the cuproptosis was inhibited in the tongue dorsal mucosa along with GIM development.

Above all, when GIM occurred, the cell landscape of tongue dorsal mucosa obviously altered. The cell death of immune cells was inhibited, such as macrophages, monocytes, T cells, and NK cells. However, cell death of non-immune cells was promoted, such as MSCs, oligodendrocytes, and SMCs, and the dead pattern included apoptosis, ferroptosis, and necroptosis.

### Pseudotime trajectory analysis and pathway enrichment analysis based on differential genes in tongue dorsal mucosa of GIM rats

**Fig. 4:**
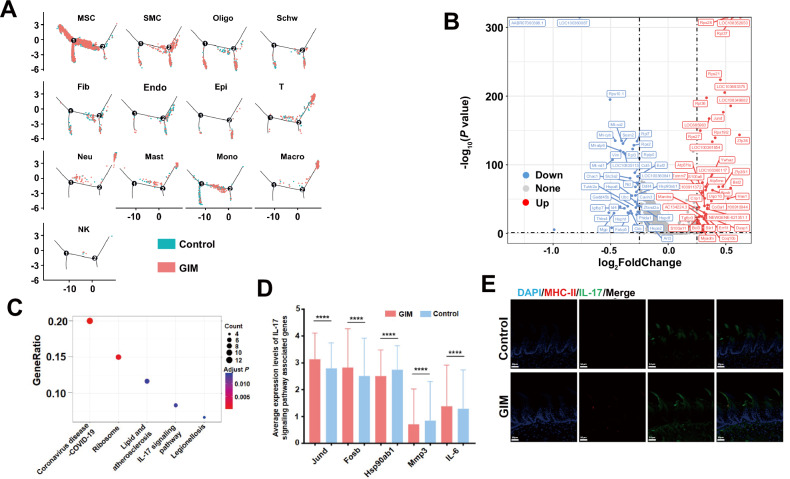
Differentiation characteristics and pathways analysis of the tongue dorsal mucosal cells in GIM rats. **A** Pseudotime analysis presented the cell distribution density along the timeline for the control group (blue) and GIM group (red) across 13 clusters. **B** Volcanic map presented the differential genes between the control and GIM groups, blue spots represented downregulating genes while red spots represented upregulating genes in GIM group. **C** KEGG enrichment analysis was conducted based on the differentially expressed genes between the control and GIM groups. **D** The differential analysis of the genes in the IL-17 pathway was conducted between the control and GIM groups. **E** Immunofluorescence staining of MHC-II molecules and IL-17 in tongue dorsal mucosa (magnification: ×20, scale bar: 50 μm). Data with error bars are shown as mean ± standard deviation. ****P < 0.0001 as determined by independent *t*-tests.

To explore the potential mechanism of tongue dorsal mucosal cell differentiation in GIM rats, pseudotime analysis revealed that the developmental trajectory of all cells comprised two differentiation nodes. Of them, MSCs distributed throughout the entire process of differentiation and development, with a predominant presence in the initial stage. SMCs, oligodendrocytes, Schwann cells, fibroblast, endothelial cells, and epithelial cells mostly assembled in the final stage of differentiation. T cells, neutrophils, mast cells, monocytes, macrophages, and NK cells were mainly distributed at both initial and final stages of differentiation (Fig. 4A). Compared with the control group, GIM rats occupied 60 upregulating genes and 53 downregulating genes in tongue dorsal mucosal tissue cells (Fig. 4B), which were mainly enriched in coronavirus-COVID-19 pathway, ribosome pathway, lipid and atherosclerosis pathway, IL-17 pathway, and legionellosis pathway (Fig. 4C). Within the IL-17 pathway, upregulating *Jund*, *Fosb*, *IL-6* and downregulating *Hsp90ab1*, *Mmp3* were observed in GIM rats (Fig. 4D). IL-6 plays a crucial role in inducing naive T cells into Th17 cells, and is also the core cytokine in promoting inflammatory response [18]. Immunofluorescence staining showed that IL-17 mainly expressed in tongue papilla and tongue papillary space, which was significantly increased in the GIM rats (Fig. 4E). The results suggested that IL-17-mediated mucosal immunity played a key role in the development of tongue dorsal mucosa in GIM rats.

Differential gene analysis was conducted on the 13 clusters between the GIM and control groups, and pathway enrichment analysis was performed based on the different genes (Fig. S4). Of them, the IL-17 pathway was enriched in MSCs and endothelial cells (Fig. S4A and S4B). Furthermore, in the IL-17 pathway, upregulating *Jund*, *Fosb*, *IL-6*, and downregulating *Hsp90ab1*, *Mmp3*, and *Cxcl2* were observed in MSCs of GIM rats (Fig. S4A). In the tongue, dorsal endothelial cells of GIM rats, *Elavl1*, *Ikbkb*, *Hsp90b1*, *Ccl7*, and *Tbk1* were significantly upregulated, while *Nfkbia* and *Hsp90ab1* were significantly downregulated (Fig. S4B). The Th17 cell differentiation pathway was notably enriched with upregulating *Il2rg*, *Fos*, *Hif1a* and downregulating *Hsp90ab1*, *Cd247*, and *Tgfb1* in the T cells of GIM rats (Fig. S4C). In epithelial cells, the differential genes were mostly enriched in the pathways related to cell proliferation, differentiation, and metabolism, such as the thermogenesis pathway, ribosome pathway, and protein processing in the endoplasmic reticulum. In addition, *Hsp90aa1* expression was significantly upregulated, while *Hspa8* and *Hspbp1* expressions were markedly downregulated in GIM rats (Fig. S4D). These findings indicated that heat shock proteins not only participated in IL-17-related inflammatory responses in the tongue dorsal mucosa of rats but also regulated the abnormal proliferation and metabolism of tongue dorsal mucosal epithelial cells.

### Cellular communication analysis in the tongue dorsal mucosa of GIM rats

**Fig. 5:**
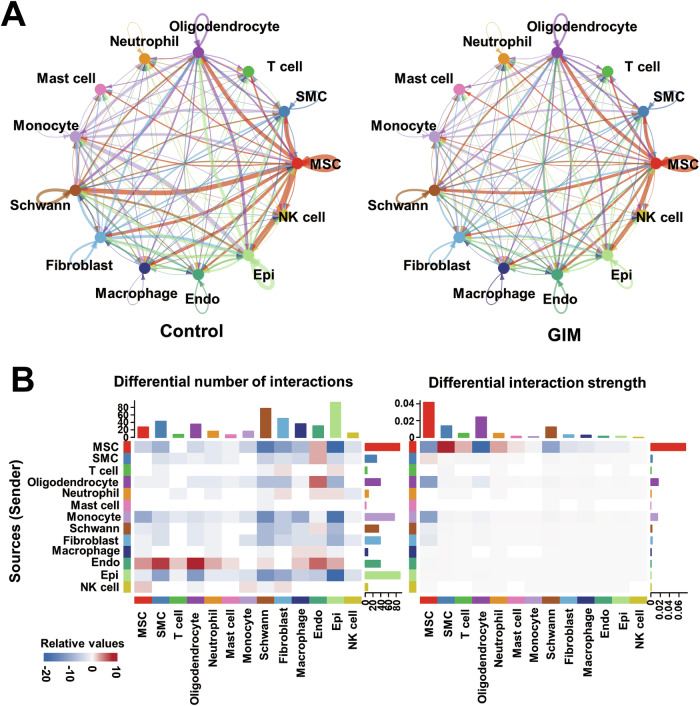
Cellular communication analysis of the tongue dorsal mucosal clusters. **A** Cellular communication visualization. Cellular communication analysis of the tongue dorsal mucosal clusters was conducted according to ligand–receptor pairs in the control group and GIM group, respectively. The arrows represent ligands, pointing to receptors, and line thickness indicates the extent of communication. **B** Based on the control group, the heatmap presented the number and intensity of cellular communications in the tongue dorsal mucosa of GIM rats. The red and blue blocks were increased and decreased, respectively. The left presents the number of communication signals, and the right presents the intensity of communication signals. The bar graph displayed the cumulative values of the heatmap rows or columns.

Cellular communication is mainly mediated by receptor–ligand pairs and plays a crucial role in coordinating various biological processes involved in cell development, differentiation, and disease. The results revealed that MSCs, monocytes, fibroblasts, and epithelial cells primarily served as signal transmitters, and oligodendrocytes served as both active signal transmitters and receivers in the control group; MSCs, oligodendrocytes, endothelial cells, monocytes, and fibroblasts predominantly functioned as signal transmitters in the GIM group (Fig. 5A). Notably, in the tongue dorsal mucosa of GIM rats, a notable reduction in cell–cell communication was observed between epithelial cells and other clusters, including MSCs, Schwann cells, and monocytes. In contrast, endothelial cells significantly increased their communication with other cells, including MSCs, SMCs, T cells, oligodendrocytes, and neutrophils. Overall, the dominant MSCs decreased their communication strength with other cells in GIM rats (Fig. 5B). The data indicated that the development of GIM shrunk the capacity of cellular communication between epithelial cells and MSCs, but enlarged the cellular communication capacity among the endothelial cells.

## Discussion

The filiform papillae, which are most abundant spreading over the tongue dorsal mucosa, mainly consist of keratinocytes. Keratinocytes undergo differentiation, migration, and keratinization, gradually transitioning from immature basal cells to stratum corneum and forming a distinctive squamous epithelium with a layered structure. Keratins serve as the key structural proteins in epithelial cells, forming intermediate filaments with a diameter of 10–12 nm to maintain mechanical stress and structural integrity [19]. Keratins exhibit different spatio-temporal expression patterns during epithelial differentiation and development, which accumulate near the membrane adhesion plaques and are rapidly expressed to maintain epithelial tissue homeostasis in case of damage and repair [20]. In this study, several keratin genes (*Krt7*, *Krt8*, *Krt13*, *Krt16*, and *Krt76*) were significantly downregulated in the tongue dorsal mucosa of GIM rats. Krt7 is a type II keratin that participates in cytoskeletal remodeling and the formation of intermediate filaments in epithelial cells, and enhances cell motility [21]. Previous studies demonstrated that *Krt7* was broadly expressed in alveolar tissues, bladders, gastric mucosa, colon mucosa, and taste buds in lingual dorsal fungiform papillae of mice. The absence of *Krt7* led to abnormal proliferation of bladder epithelial cells in mice [22], and its expression positively correlated with the degree of sclerosis of the keratinized epithelium [23]. *Krt8* is a marker of taste bud cells in mouse tongue dorsal mucosa [24]. *Krt76* knock-out mice had swollen spleens and lymph nodes, had higher levels of serum pro-inflammatory cytokines (IL-6, IL-10, and TNF-α), and more regulatory T cells appeared in lymph nodes [25]. These findings indicated that deoxycholate-induced GIM rats exhibited weakened taste sensitivity, reduced hardening of keratinocytes in the tongue dorsal mucosa, impaired integrity and stability of the tongue dorsal mucosal epithelium, as well as heightened inflammatory activity in the basal layer of the tongue dorsal mucosa.

Oral mucosa locates the entrance of the mucosal barrier of the digestive tract, and the IL-17/Th17-mediated immune response plays a pivotal role in maintaining the integrity of the mucosal barrier and preventing oral infections [26]. The present study found that the IL-17 signaling pathway was enriched in the tongue dorsal mucosa of GIM rats. The upregulating *IL-6* in MSCs and upregulating *Hsp90aa1* epithelial cells indicated the active inflammatory response in the tongue dorsal mucosa. IL-6 is known for inducing naive T cells to differentiate into Th17 cells, thereby amplifying inflammatory responses [18]. This process is in line with the observed upregulating IL-6, which is likely a consequence of the inflammatory activity triggered by the downregulation of Krt76. Additionally, Hsp90 functions as a critical chaperone for proper protein folding, its overexpression promotes the proliferation and metastasis of gastric cancer cells [27], and inhibiting Hsp90 is an important anti-tumor strategy [28]. The results confirmed the inflammatory activity and abnormal epithelial cell proliferation in the tongue dorsal mucosa of GIM rats.

The shedding and renewal of epithelial cells are essential for maintaining the functional homeostasis of the skin and mucosa. This study observed many marker genes involved in several classic types of cell death in the tongue dorsal mucosal cells of GIM rats. For example, the autophagy-related gene *Map1lc3* was significantly downregulated in both neutrophils and T cells. Previous studies demonstrated that autophagy played a pivotal role in eliminating dying cells, reducing inflammasome formation, and limiting inflammatory activity [29]. Clinical evidence showed that the patients with coronary atherosclerosis had lower Map1lc3 expression in peripheral leukocytes [30], and autophagy gene defect in macrophages led to excessive activation of inflammasomes and overexpression of IL-1β [31]. Thus, significant downregulating *Map1lc3* and upregulating *IL-1β* in T cells and neutrophils might be the essential driving force of increasing infiltration of submucosal immune cells. As regards necroptosis, the lower *Ripk3* expression was observed in macrophages of tongue dorsal mucosa in GIM rats. *Ripk3* is a crucial gene in necroptosis by participating in cellular reactive oxygen species accumulation, promoting fatty acid oxidation and inducing the polarization of M2 macrophages in the tumor microenvironment [32]. It suggested that the M2 phenotype was inhibited in the tongue dorsal mucosa of GIM rats. In addition, *Dlst*, a cuproptosis marker gene, was significantly downregulated in fibroblasts in the GIM rats. Previous studies demonstrated that the transform of fibroblasts was closely related to cuproptosis, the cuproptosis was inhibited during the transition of quiescent fibroblasts into myofibroblasts [33]. Similarly, the decreasing *Dlst* and *Pdha1* were observed in the fibroblasts within hypertrophic scar tissue [34]. The results suggested that the inhibited cuproptosis might damage the integrity of the tongue dorsal mucosal barrier in GIM rats.

As well as known, the tongue functions taste sensation, which depends on various receptors spreading over the tongue dorsal mucosa. Of them, the salty and sour receptors are ion channels, while the bitter, sweet, and umami receptors are G-protein-coupled receptors [35]. The present study observed the downregulating bitter receptor Rtp4 and sweet receptor Tas1r2 in the tongue dorsal mucosa of GIM rats. Previous literature showed that the *Rtp4* expression in melanoma tissue was significantly positively correlated with neutrophil infiltration, and higher *Rtp4* expression foreshadowed longer overall survival of melanoma patients [36]. The sweet taste receptor is a heterodimer formed by Tas1r2 and Tas1r3, and the inflammation could cause obvious taste disorders. Clinical investigations showed that abnormal taste function was the common symptom in patients with respiratory viral infections, oral infections, or viral hepatitis [37–39]. The animal experiment found that the mice with lupus erythematosus had a reduced preference for sucrose along with significantly increased serum levels of IL-6 and interferon-γ [40], and downregulating Tas1r2 was observed in the mice with chronic colitis disease [41]. These evidences suggested that inflammatory response could decrease the perception of bitter and sweet tastes in the tongue dorsal mucosa of GIM rats.

To sum up, this study comprehensively depicted the cell landscape of the tongue dorsal mucosa in GIM rats. The main findings are as follows: (1) The inflammatory activity might link with the downregulating *Krt76* in the tongue dorsal mucosa of GIM rats; (2) The regulated cell death of immune cells was inhibited, while the regulated cell death of non-immune cells was promoted; (3) The tongue mucosal inflammatory activity might lead to the weakened sensation of bitter and sweet in GIM rats. There are still limitations in this study, as utilized the scRNA-seq method, which generally has a lower sequencing depth compared to RNA-seq of bulk tissues, and these findings need further behavioral experiments and in vitro cell experiments to disclose the underlying mechanism. Anyway, this study partly interpreted the parageusia and abnormal tongue dorsal mucosa (tongue coating) in patients with gastric precancerous lesions. The present data provided a fresh insight into the linkage between tongue diagnosis and gastrointestinal disorders, at least the bile reflux-induced precancerous lesions of gastric cancer.

## Materials and methods

### Preparation of the rat model with GIM

According to the previous study [42], specific-pathogen-free (SPF) male Sprague–Dawley (SD) rats aged 5–6 weeks (Zhejiang Weitong Lihua Experimental Animal Technology Co., Ltd., 150-190 g) were randomly divided into control group (*n* = 5) and model group (*n* = 6). The control group was orally gavaged with 2 mL of sterile water daily, while the model group was orally treated with 2% sodium salicylate solution (Beyotime Biotech. Inc., China, No. S3007) in 2 mL, freely drank 20 mmol/L of deoxycholate sodium (Beyotime Biotech. Inc., Shanghai, China, No. ST2049) every day. When the model rats were diagnosed with GIM by histopathological analysis after 12 weeks, and the control and GIM rats were collected for scRNA-seq of tongue dorsal mucosa. This study was approved by the Animal Ethics and Protection Committee of Nanjing University of Chinese Medicine (Nanjing, China; No. 202102A004).

### Preparing single-cell suspension of tongue dorsal mucosa

The rat tongue (*n* = 3 in each) was collected from the root of the tongue, and the tongue was divided into upper and lower parts along the lingual alveoli. The tongue dorsal mucosa was extracted after carefully removing the muscle tissue from the upper part as soon as possible. The tongue dorsal mucosa was washed with phosphate-buffered saline (PBS) and was minced into small pieces (~1 mm^3^) on ice. The single-cell suspension was prepared by enzymatical digestion solution including 100 U/mL collagenase I (Worthington, LS004217), 100 U/mL collagenase IV (Worthington, LS005288), and 30 U/mL DNase I (Worthington, LS002139). The digestive system was incubated in a 37 °C shaking water bath for 45 min and the degree of cell dissociation was checked every 10 min.

The single-cell suspension was sieved through a 70 µm cell strainer (Biosharp, BS-70-XBS), and centrifuged at 400×*g* for 5 min; the pelleted cells were suspended in red blood cell lysis buffer (Beyotime, C3702) to remove the red blood cells. After washing with PBS (Beyotime, C0251) containing 0.04% BSA, the cell pellets were re-suspended in PBS containing 0.04% BSA and re-filtered through a 40 μm cell strainer (Biosharp, BS-40-XBS). Finally, cells were marked with DRAQ7 (Abcam, AB109202) and Calcein AM (Thermo Fisher Scientific, C1430), which are used for cell activity detection. The quality of cell suspensions was judged by a fluorescence cell analyzer (Countstar Rigel S2, Countstar).

### Single-cell transcriptome sequencing

The BD Rhapsody system (Becton, Dickinson and Company, Franklin Lakes, New Jersey, USA) was used to conduct single-cell transcriptome sequencing of tongue dorsal mucosal cells. Briefly, the suspension was randomly distributed across more than 200,000 microwells by a limited dilution approach. Beads containing oligonucleotide barcodes were required to be added excessively to make sure that almost every microwell has one bead. Once the cells are captured, the lysis buffer (Thermo Fisher Scientific, cat. 4458235) goes into effect. The chemical components in the lysis buffer disrupt the cell membrane and nuclear membrane, allowing the intracellular RNA molecules to be released. As the cell membrane ruptures, mRNA molecules are released into the microwell solution. These mRNA molecules then hybridize with the oligonucleotides on the barcode capture beads, assigning a unique barcode identifier to each mRNA molecule. Beads were collected into a single tube for reverse transcription and ExoI digestion. Then, the unique molecular identifier (UMI) and cell barcode were used to tag the synthetic cDNA at the 5’ end, which is equivalent to the 3’ end of the mRNA molecule. Finally, the single-cell library was generated through several procedures, including random priming and extension (RPE), RPE amplification PCR, and WTA index PCR. Library quantification was performed by Agilent Bioanalyzer 2200 High Sensitivity DNA Chip and Qubit High Sensitivity DNA assay (Thermo Fisher Scientific). All generated libraries were sequenced by an Illumina sequencer (Illumina, San Diego, CA) with PE150 strategy (paired-end 150 bp).

### Cell filtration and quality control

The raw gene expression matrix was generated using CellRanger (V 1.1.0), and convert them into Seurat objects using the Seurat R package (V 4.3.0). Low-quality cells were filtrated with the following criteria in the single cell: (i) >3000 or <200 genes detected; (ii) mitochondrial gene percentage >40%. Then, the gene expression matrix was normalized by the NormalizeData function, and 2000 features with high cell-to-cell variation were calculated using the FindVariableFeatures function. To reduce the dimensionality of the datasets, the RunPCA function was conducted with default parameters on scaled data generated by the ScaleData function.

### Cluster analysis and identification

After nonlinear dimensional reduction and projection of all cells into two-dimensional space by uniform manifold approximation and projection (UMAP), cells clustered together according to common features. The FindAllMarker function in the Seurat package was utilized to identify marker genes for each identified cluster. Cell clusters were annotated based on the marker gene expressions.

### Distribution of cell cycle

Based on the relative expression of G2/M and S phase-related gene sets (Table S1), the CellCycleScoring function was used to determine the stage of the cell cycle for each cell.

### Differential analysis of key genes

The key genes related to cell keratinization, taste receptors, and cell death were analyzed for differential gene expression between the control and the GIM groups using the AverageExpression function, and the expression level (mean ± SD) of each gene was calculated using GraphPad Prism v9 (GraphPad Software Inc., San Diego, CA, USA), and displayed using ggplot2.

### Pseudotime analysis

The dominant cells in tongue dorsal mucosa were mesenchymal stem cells, T cells, epithelial cells, and neutrophils, and their developmental trajectories were analyzed by Monocle 2 (V 2.26.0).

### Gene function enrichment analysis

The differential expression of mesenchymal stem cells, T cells, and epithelial cells was analyzed using FindMarkers function between control and GIM groups. Genes with *P* value 0.05 and log2-fold change (FC) absolute value >0.25, were selected as the candidate genes for KEGG pathway enrichment analysis, which was conducted using org.Rn.eg.db package and clusterProfiler (V 4.7.1.3) package.

### Cell–cell communication analysis

According to previous literature [43], intercellular communication analysis was conducted using CellChat in the control and GIM groups, respectively. Based on the ligand–receptor pattern, the number and intensity of cell interactions were demonstrated among the cell clusters to explore the potential cell communication patterns in the tongue dorsal mucosa.

### Histopathological analysis

The tongue tissue of control group (*n* = 5) and GIM group (*n* = 6) was fixed with 4% paraformaldehyde, embedded in paraffin, and cut into 5 μm sections. After routine dewaxing and hydration, hematoxylin and eosin (H&E) staining was performed strictly according to the instruction of H&E Staining Kit (Servicebio, G1005).

Paraffin dehydrated tissue sections were placed in antigen repair buffer (Servicebio, G1206, pH 8.0) for antigen retrieval in a microwave oven (Galanz, D4B0-S2) at medium power for 8 min, followed by a cooling period of 8 min, and then at low-medium power for 7 min. To block the endogenous peroxidase activity, the slices were put into 3% hydrogen peroxide and incubated at room temperature in darkness for 25 min. Antigen was then blocked with 3% BSA (Servicebio, GC305010) for 30 min. Subsequently, tissue sections were incubated with primary antibodies at 4 °C overnight, washed three times with PBS, and incubated with CY3 and IgG+HRP labeled secondary antibodies at 37 °C for 50 min. The information of six primary antibodies were as following: Krt76 (BS-1005R, diluted 1:200; Beijing Boaosen, China), CD45 (GB113886, diluted 1:2000; Servicebio, China), HLA-DB (DF6408, diluted 1:200; Affinity Biosciences, America), IL-17 (GB11110-1, diluted 1:2000; Servicebio, China), Mapl1c3a (BM4367, diluted 1:200; Boshide, China), and Gpx4 (GB114327, diluted 1:2000; Servicebio, China).

The slides were washed three times in PBS, air-dried slightly, and then subjected to a Tyramide Signal Amplification (TSA) staining kit (Servicebio, G1231). Here, repeat the above step for the second antigen staining. Finally, the slides were air-dried, followed by the addition of DAPI staining solution (Servicebio, G1012) for nuclear counterstaining, washed with PBS 3 times, and autofluorescence quencher B (Servicebio, G1221) was added for 5 min. The anti-fluorescence quencher (Servicebio, G1401) was used to seal the slides, and the panoramic images were acquired using a fluorescence microscope (Nikon Eclipse C1, Nikon, Tokyo, Japan) and a Pannoramic MIDI scanner (3DHISTECH Ltd., Budapest, Hungary).

### Statistical analysis

All statistical analyses were performed using GraphPad Prism v9. Normally distributed data were presented as mean ± standard deviation and compared using independent *t*-tests. *P*-value < 0.05 was used to indicate significance. *P*-values are indicated as follows: *P < 0.05, **P < 0.01, ***P < 0.001, and ****P < 0.0001.

## Supplementary information

Supplementary materials, including supplementary figures and legends.

Supplementary materials, including supplementary tables.

## Data Availability

The raw sequence data have been deposited in the Genome Sequence Archive in National Genomics Data Center, China National Center for Bioinformation/Beijing Institute of Genomics, and Chinese Academy of Sciences (GSA: CRA017434) that are publicly accessible at https://ngdc.cncb.ac.cn/gsa.

## Code availability

The source code used to analyze the scRNA-seq data in the current study is available online at the GitHub repository (https://github.com/BananaFu-bit/Tongue-Dorsal-Mucosa-in-Rats.git).
