# Supplementary material for: Single-cell RNA sequencing revealed cell landscape of tongue dorsal mucosa in rats with gastric intestinal metaplasia
Source: Cell Death Discov. 2025 Mar 16;11:105. doi: 10.1038/s41420-025-02386-z (PMC11911441; doi:10.1038/s41420-025-02386-z)
Supplement: Supplementary file 1 — Supplementary materials, including supplementary figures and legends. [file 41420_2025_2386_MOESM1_ESM.pdf]

Supplement Figure 1.

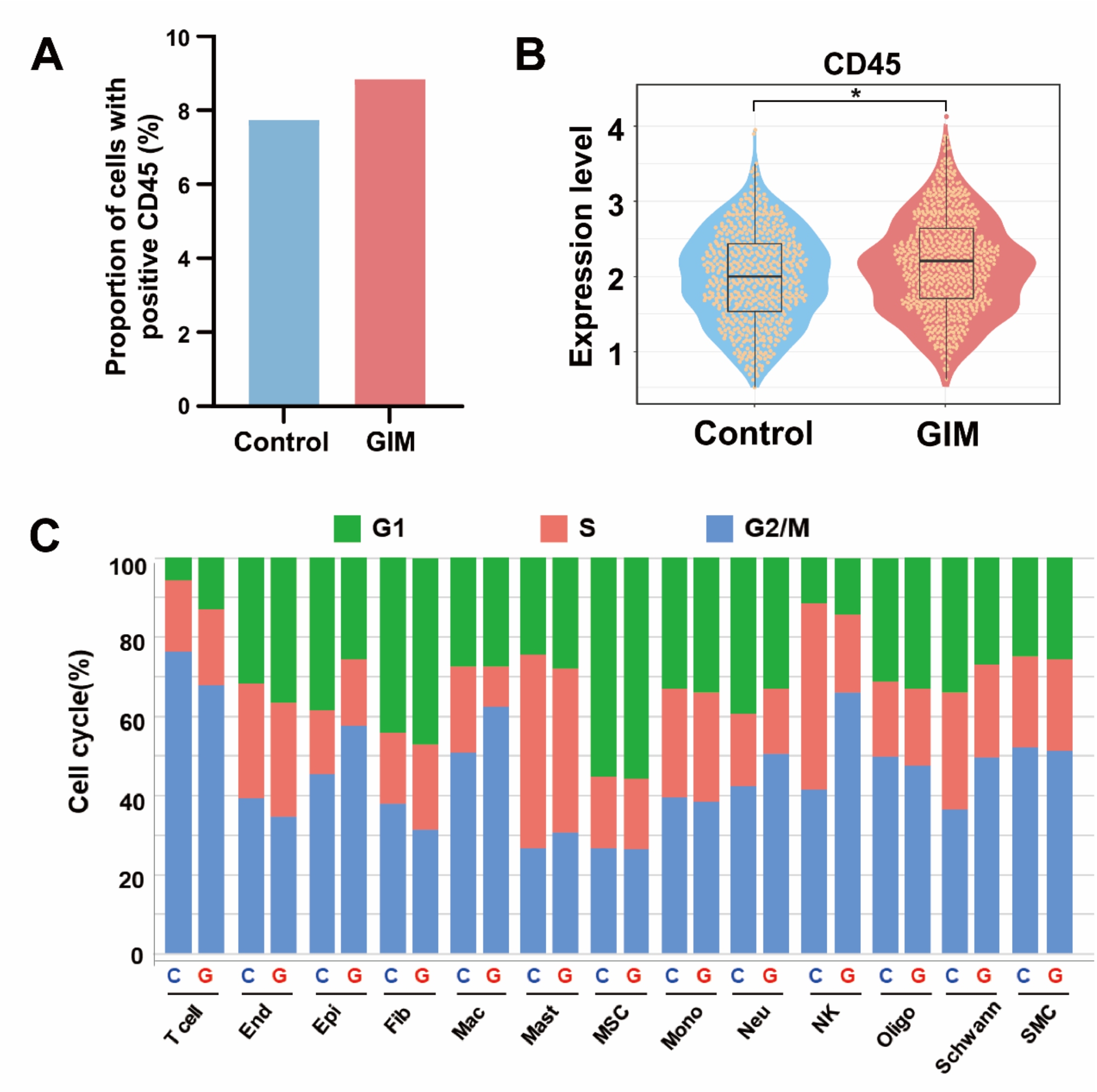

Cell cycle distribution of tongue dorsal mucosa in GIM rats. A The percentage of CD45-positive cells in tongue dorsal mucosa of GIM rats. **B** Expressive level of CD45 in tongue dorsal mucosa of GIM rats, data with error bars are shown as mean ± standard deviation. *$P < 0.05$ as determined by independent t-tests. **C** Cell cycle distribution in tongue dorsal mucosa of GIM rats.

Supplement Figure 2.

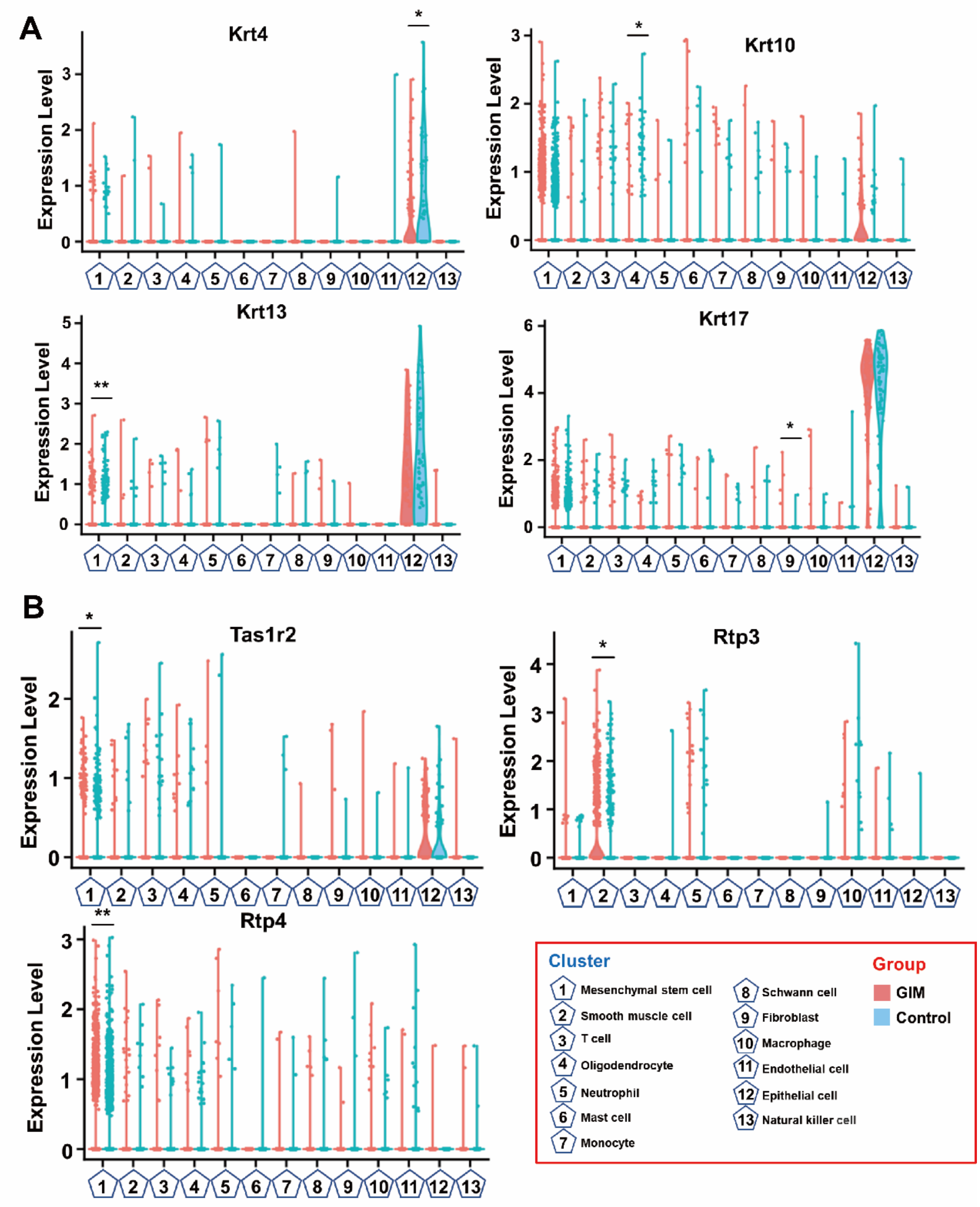

Differential analysis of keratin and taste receptor genes in clusters of tongue dorsal mucosa in GIM rats. A Four differential Krt genes were exhibited in the 13 clusters of tongue dorsal mucosa. **B** Three taste sensing genes were of significant differences in the 13 clusters of tongue

dorsal mucosa. Data with error bars are shown as mean ± standard deviation. $^{*}P < 0.05$; $^{**}P < 0.01$ as determined by independent t-tests.

Supplement Figure 3.

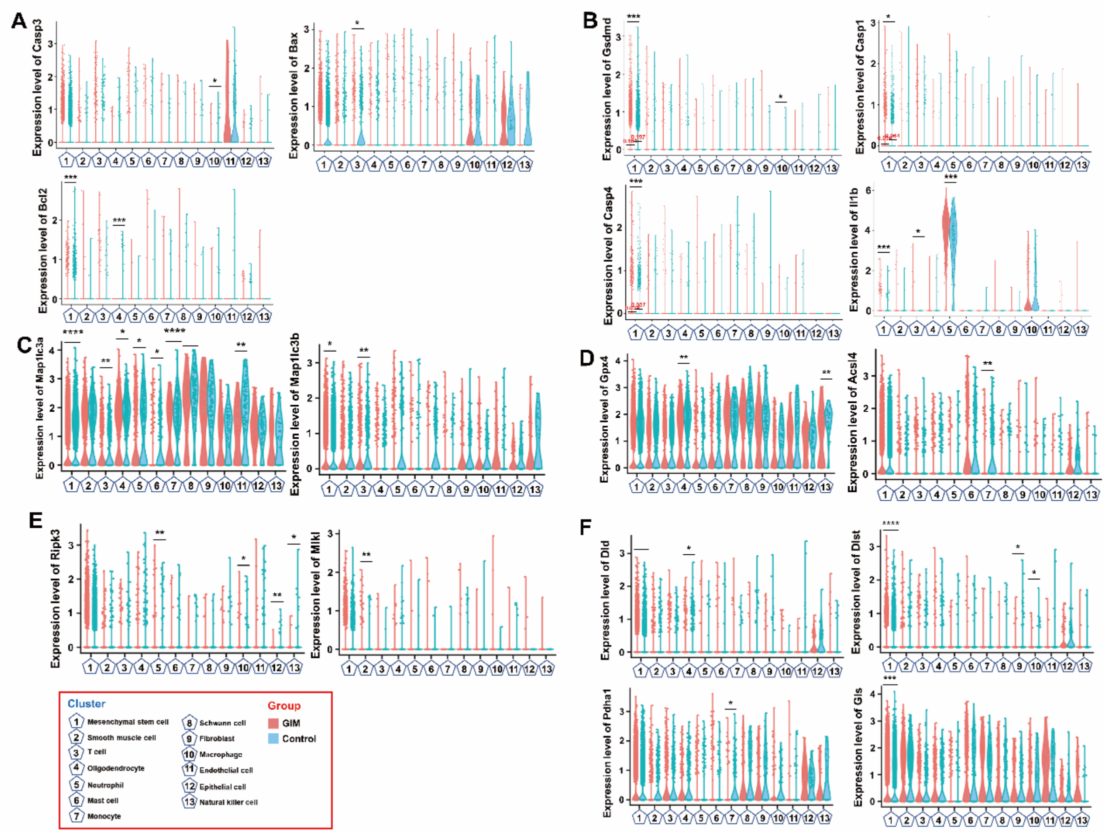

Differential analysis of cell death genes expression in the 13 clusters of the tongue dorsal mucosa of GIM rats. Stratified differential analysis of the cell-death-related genes in the 13 clusters between the control and GIM groups. **A** Apoptosis-related genes, **B** pyroptosis-related genes, **C** autophagy-related genes, **D** ferroptosis-related genes, **E** necroptosis-related genes, and **F** cuproptosis-related genes. Data with error bars are shown as mean ± standard deviation. *P < 0.05, **P < 0.01, ***P < 0.001, and ****P < 0.0001 as determined by independent t-tests.

Supplement Figure 4.

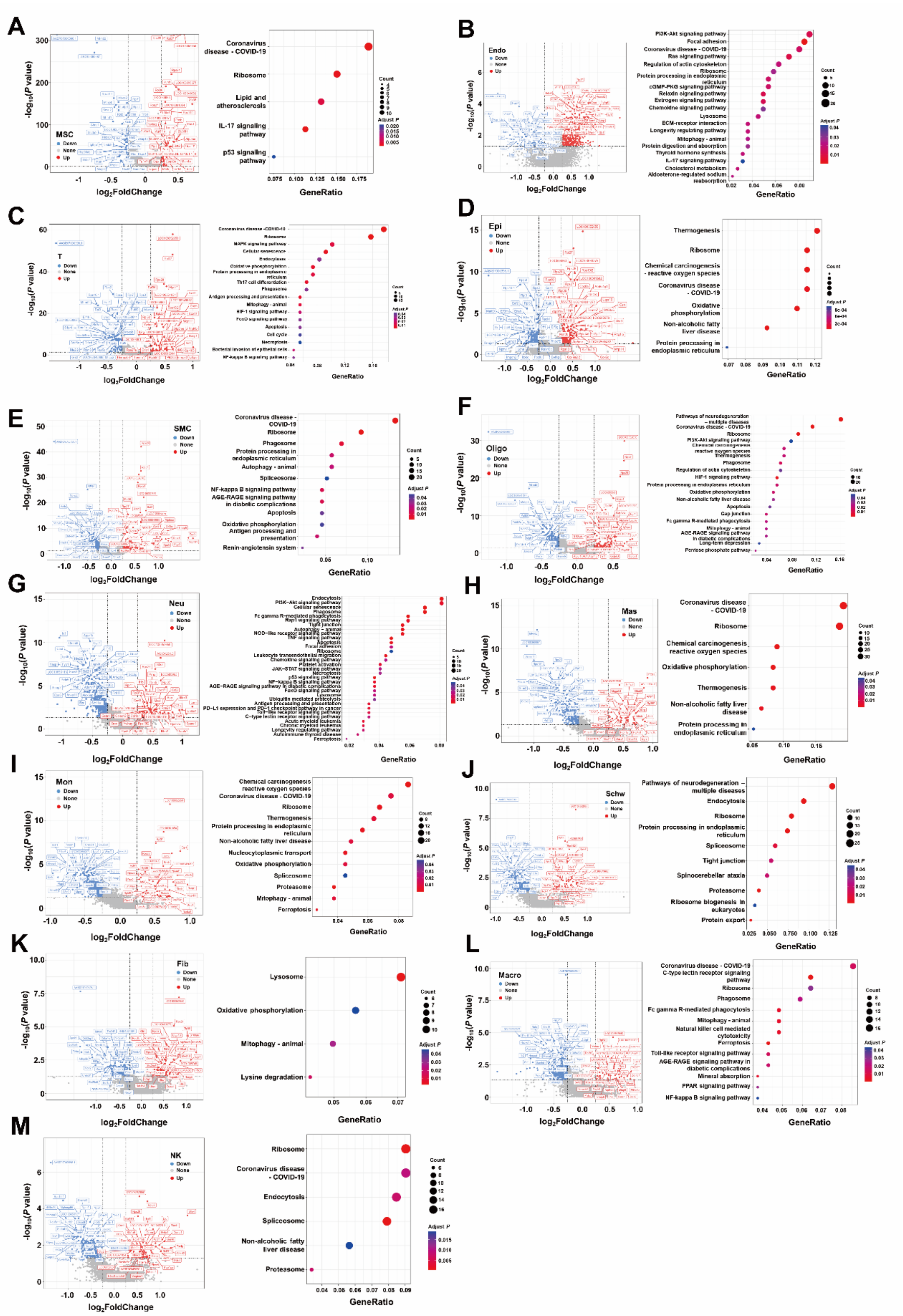

Differential analysis of genes and KEGG pathway enrichment

analysis in the 13 clusters of tongue dorsal mucosa in GIM rats. Differential analysis of genes in the 13 clusters between the control and GIM groups, and KEGG pathways were enrichment based on the different genes. **A-M** volcano plot and KEGG enrichment bubble chart for differential genes in the 13 clusters: **A** mesenchymal stem cells, **B** endothelial cells, **C** T cells, **D** epithelial cell, **E** smooth muscle cells, **F** oligodendrocytes, **G** neutrophil, **H** mast cells, **I** monocytes, **J** schwann cells, **K** fibroblasts, **L** macrophages, **M** NK cells.

Supplement Tables

Table S1. The differentially expressed genes in the 13 clusters

Table S2. Marker genes of S and G2/M phages

Table S3. Cell cycle distribution of the 13 clusters in control and GIM groups

Table S4. Differential analysis of keratin genes between control and GIM groups

Table S5. Differential analysis of keratin genes in the 13 clusters between control and GIM groups

Table S6. Differential analysis of gustatory genes between control and GIM groups

Table S7. Differential analysis of gustatory genes in the 13 clusters between control and GIM groups

Table S8. Differential analysis of the marker genes in the six types of cell death between control and GIM groups

Table S9. Differential analysis of apoptosis-related genes in the 13 clusters between control and GIM groups

Table S10. Differential analysis of pyroptosis-related genes in 13 clusters between control and GIM groups

Table S11. Differential analysis of autophagy-related genes in 13 clusters between control and GIM groups

Table S12. Differential analysis of ferroptosis-related genes in 13 clusters between control and GIM groups

Table S13. Differential analysis of necroptosis-related genes in 13 clusters between control and GIM groups

Table S14. Differential analysis of cuproptosis-related genes in 13 clusters between control and GIM groups
